# Age is associated with unfavorable neuropathological and radiological features and poor outcome in patients with WHO grade 2 and 3 gliomas

**DOI:** 10.1038/s41598-021-96832-4

**Published:** 2021-08-30

**Authors:** Aleksandrs Krigers, Matthias Demetz, Claudius Thomé, Christian F. Freyschlag

**Affiliations:** Department of Neurosurgery, Medical University Innsbruck, Innsbruck, Austria

**Keywords:** Cancer epidemiology, CNS cancer

## Abstract

With the rising life expectancy and availability of neuroimaging, increased number of older patients will present with diffuse and anaplastic gliomas. The aim of our study was therefore to investigate age-related prognostic clinical, neuropathological and radiological features of lower-grade gliomas. All consecutive patients with diffuse or anaplastic glioma WHO grade 2 and 3 who underwent first tumor resection between 2010 and 2018, were selected from the institutional neuro-oncological database and evaluated. The mean age of 55 males and 44 females was 46 years (SD ± 16). Wild-type IDH1 (p = 0.012), persistent nuclear ATRX expression (p = 0.012) and anaplasia (p < 0.001) were significantly associated with higher age. The CE volume before resection was found to be increased in older patients (r = 0.42, p < 0.0001), and CE rate was higher in the IDH wild-type population only (p = 0.02). The extent of resection did not differ with age. Overall, one year of life resulted in a PFS reduction of 9 days (p = 0.047); in IDH sub-group analysis, this dependency was confirmed only in wild-type tumors (p = 0.05). OS was significantly reduced in older patients (p = 0.033). In conclusion, behavior and prognosis of WHO grade 2 and 3 glioma were unfavorable in correlation to patient’s age, even if the extent of resection was comparable. Older age imparted a poorer PFS and higher CE rate only in the IDH wild-type population.

## Introduction

Gliomas are the most common primary malignant brain tumors and can be found in all age groups^1,2^. Patients with lower-grade gliomas of WHO grade 2 and 3 (LGG) are usually younger than those harboring a glioblastoma WHO grade 4^3^ and commonly have a more favorable course. Nevertheless, LGGs often exhibit malignant degeneration further limiting overall survival^4^. Nowadays, the influence of genetic features is considered to be more important than the phenotype or WHO grade alone^5^. Patients with IDH-wild type anaplastic astrocytomas were found to show worse outcome than IDH mutated glioblastomas^6^.

The absolute number of older patients diagnosed with WHO grade 2 and 3 gliomas will increase in the future due to rising availability of neuroimaging^7^ in combination with the advancing age of the general population. The proportion of people over 65 in the European Union is expected to increase from 31.4% in 2019 to 52.0% in 2050 ^8^. This can be translated to all industrialized countries.

The initial treatment of choice is complete resection of the tumor^9–11^. The extent of resection in older patients with gliomas has been reported to be lower in an attempt to minimize neurological deficits and thus avoid secondary age-related complications. Not surprisingly, overall survival in this cohort was below the published median^12–14^. Preoperative tumor volumes, molecular characteristics and/or the recent WHO classification, however, have often not been taken into account in elderly glioma patients. Moreover, variable age cutoffs between 55 and 65 years have been used^14–16^, which does not reflect contemporary observations of increased disability-free life years according to the changed general life expectancy^17,18^. Thus, previous data may not affect the current trends of aged populations in neuro-oncology.

The aim of our study was therefore to investigate age-related prognostic features in terms of clinical, neuropathological and radiological examinations of diffuse and anaplastic gliomas WHO grade 2 and 3. A cut-off for age was avoided to determine the effect of each additional year of life.

## Materials and methods

All consecutive adult patients with neuropathologically proved intracranial WHO grade 2 and 3 gliomas, operated in our center for their first resection between January 2010 and March 2018, were selected from our prospective neurooncological database. The study was approved by the ethics committee of Medical University Innsbruck (AN5220 329/4.4) and written informed content was acquired from all participants. This study was performed in accordance with the ethical standards as laid down in the 1964 Declaration of Helsinki and its later amendments or comparable ethical standards.

Preoperative and postoperative MRI within 72 h were performed including T1-weighted Gadolinum-contrasted as well as native T1, T2, FLAIR and DWI sequences as the standard of care for patients harboring intracranial tumors^19^. The metric volume of alteration was manually measured using segmentation in ITK-SNAP software (v.3.8.0 for Mac OS, UPenn and UNC dev., http://www.itksnap.org) in T1 with contrast enhancement (CE) as well as native T1, T2, FLAIR and DWI sequences^20,21^.

Neuropathological examination was routinely performed on FFP-embedded tissue. Integrated neuropathological reports were based on the WHO grading system^22,23^. Presence of IDH1 mutation in the R132H position was assessed with IHC and, in case of negative result, DNA sequencing was performed for patients under 40 years to approve the IDH1 wild-type status. Nuclear ATRX and EGFR expression were tested with IHC. In case of lost ATRX, the oligodendroglial genotype was proved with the unbalanced co-deletion of 1p/19q chromosomal regions through Fluorescence in situ hybridization (FISH).

After the final neuropathological conclusion, each case was individually discussed in the institutional multidisciplinary tumor-board to establish the adjuvant management. The standardized recommendation was based on international guidelines^10,11,24,25^. In case of higher risk tumors – anaplastic glioma, incomplete resection, wild-type IDH1 or lost nuclear ATRX – the radio-chemotherapy according to previously published protocols^11,26^ was applied; and if the mentioned higher risk tumor criteria were absent, the wait and see strategy was chosen.

Statistical analysis and graphics generation was processed using IBM SPSS Statistics (IBM SPSS Statistics for Mac OS, Version 26.0. Armonk, NY: IBM Corp., https://www.ibm.com/analytics/spss-statistics-software). For analysis, patients were divided in two groups according to the median age: younger than median and older than median. Normal distribution of scale parameters was checked by the Kolmogorov–Smirnov test. Correlations for non-parametric data were determined using the Spearman’s method. T-Tests for normal distributed scale parameters, Mann–Whitney U-test for rank and scale parameters lacking normal distribution, and Chi^2^-test comparing two binominal parameters were applied according to general terms of these tests. The linear and Cox regressions were used to reveal the progression free survival (PFS) and overall survival (OS) dependencies. The Holm-Bonferroni (H-B) correction was corrected for multiple hypothesis^27,28^. The confidence interval and α were defined as 95%.

### Ethics approval

The study was performed in accordance with the ethical standards as laid down in the 1964 Declaration of Helsinki and its later amendments or comparable ethical standards. The database and tissue bank are approved by the ethics committee of Medical University Innsbruck (AN5220 329/4.4).

### Consent to participate

Written informed content was acquired from the participants.

### Consent to publication

No individual data is showed separately in the manuscript. All data is used only after anonymized statistical processing and is described with pool results.

## Results

In total, 99 patients were eligible for the analysis: 55 (56%) males and 44 (44%) females with a mean age of 46 years (SD ± 16; range 18–85). Patients harboring more aggressive tumors were older: 47 patients with diffuse glioma had a mean age of 40 years (SD ± 14) compared to 51 years (SD ± 16) in 52 patients with anaplastic transformed gliomas (p < 0.001, H-B: 0.003). IDH1 was mutated in 71% and wild-type in 29% of cases. ATRX was expressed in 62% and lost in 38% of LGG samples.

Patients with IDH1 mutation were found to be younger compared to those with wild-type: 43 years (SD ± 13) vs. 54 years (SD ± 18) respectively (p = 0.008, H-B: 0.012). That was also the case for the loss of nuclear ATRX expression: 39 years (SD ± 11) vs. 51 years (SD ± 16) respectively (p = 0.006, H-B: 0.012).

CE was present in 26% (13/50 of < 45y.) of cases in younger group and in 63% (31/49 of ≥ 45y.) of cases in older group (p < 0.001). Patients with higher age, who had IDH wild-type glioma, showed CE more frequently (p = 0.02); on the other hand, we could not find analogous dependency in case of IDH-mutated tumors (p = 0.11). CE volume before resection directly correlated with age, while the native volumes did not show differences (Table 1).

**Table 1:** Median tumor volume in various MRI sequences before resection.

	T1 CE, cm^3^	T1 native, cm^3^	T2 native, cm^3^	FLAIR, cm^3^	DWI, cm^3^
Younger 50% (< 45y.)	0.0 (IqR: 0.0–0.4)	30.8 (IqR: 9.4–64.1)	49.0 (IqR: 14.4–83.1)	31.3 (IqR: 13.1–82.5)	45.7 (IqR: 15.2–84.3)
Older 50% (≥ 45y.)	0.4 (IqR: 0.0–6.7)	26.6 (IqR: 11.3–42.7)	37.4 (IqR: 17.0–79.7)	40.2 (IqR: 17.7–83.1)	30.0 (IqR: 14.0–63.6)
Spearman, r	0.42	–	–	–	–
Spearman, p	0.00001	0.63	0.98	0.54	0.41
Holm–Bonferroni, p	< 0.0001	n.s	n.s	n.s	n.s

There were no differences in the extent of resection in relation to age. Complete resection of contrast enhancing tumor parts, if present, was achieved in 88% compared to 74% (younger vs. older; p > 0.05). Thus, 96% patients of younger group and 93% of older group did not have any CE after resection. The volume of residual CE tumor, however, correlated with age; r = 0.27, p = 0.013. The probability of gross total resection for FLAIR (83% vs. 78%) and DWI (85% vs. 78%) was similar between our age-related groups, p > 0.05.

**Figure 1:**
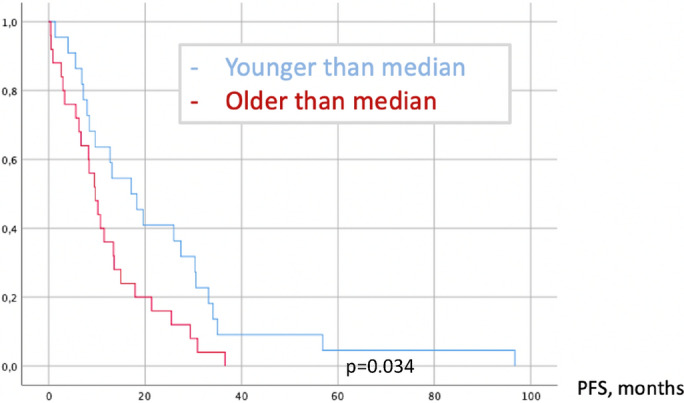
Achieved PFS in relation to age.

**Figure 2:**
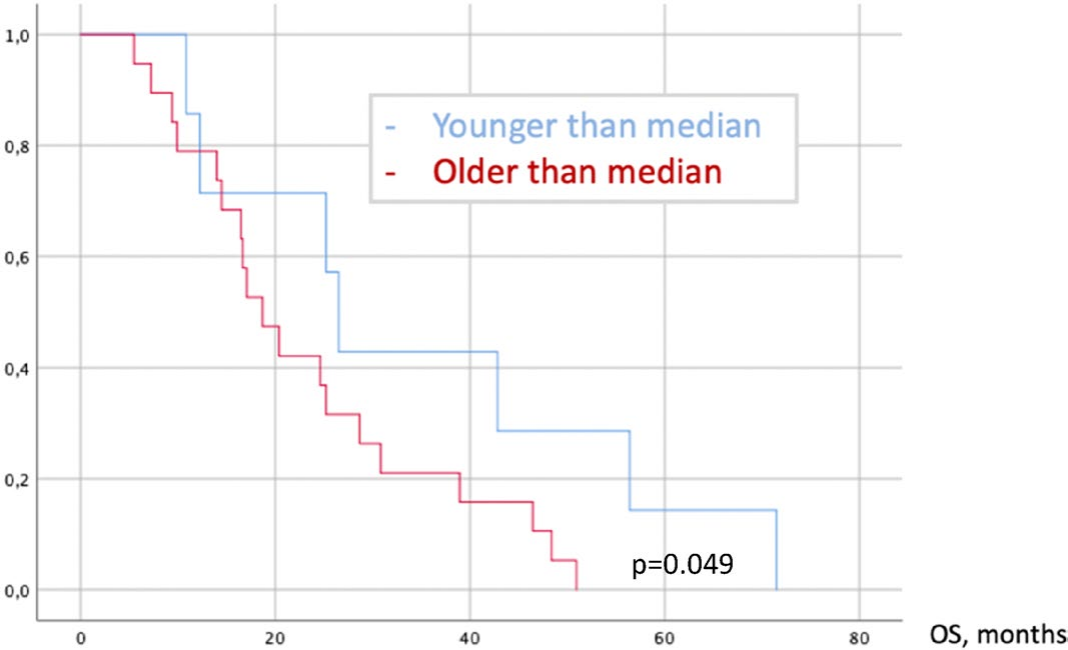
Achieved OS in relation to age.

Mean follow-up was 54 months (SE ± 32; range 5–115) in our series, whereas 47 (47%) patients reached oncological progression (Fig. 1) and 26 (26%) deceased (Fig. 2). PFS in older patients was significantly lower according to Cox regression: HR = 1.032 per year of life (CI95% 1.012–1.051; p = 0.001). One year of life reduced PFS for 9 days (0.29 months) in linear regression:

$$d{\text{PFS}} = - 0.29 \times {\text{Age}}$$

$$[{\text{change of PFS in months}} = - 0.29\,\left( {{\text{SE}} \pm 0.14} \right) \times {\text{age in years}}\;({\text{ANOVA p}} < 0.001,\;{\text{p}} = 0.047)]$$

We stratified our cohort in relation to IDH1 status. In case of wild-type IDH1, PFS by older patients was significantly lower according to Cox regression: HR = 1.031 per year of life (CI95% 1.000–1.063; p = 0.05). The significance level in the linear regression was not reached: the trend was PFS reduction for 7 days (0.24 months) per year of life:

$$d{\text{PFS}} = - 0.24\,\times\,{\text{Age}}$$

$$\left[ {{\text{change of PFS in months}} = - 0.24\,\left( {{\text{SE}} \pm 0.18} \right) \times {\text{age in years}}\; \left( {{\text{ANOVA p}} = 0.021,{\text{p}} = 0.2} \right)} \right]$$

If IDH1 mutation was present, we could not confirm the PFS association with age in Cox regression (HR = 1.010; CI95% 0.979–1.052; p – n.s.) or linear regression (p – n.s.).

OS was significantly poorer in older patients according to Cox regression: HR = 1.040 per year of life (CI95% 1.003–1.079; p = 0.033); in sub-group analysis considering IDH1 status, no significant dependencies were found (p – n.s.).

## Discussion

Older patients with diffuse and anaplastic WHO grade 2 and 3 gliomas had higher CE volume and more frequently harbored negative prognostic molecular markers like wild-type IDH. Although resection rate was similar, the residual CE volume was higher in older population. Overall, one year of patient’s life reduced the PFS by 9 days and OS was significantly decreased in relation to age. Moreover, in IDH sub-group analysis, age-dependent PFS reduction as well as higher CE rate in MRI was confirmed only in case of wild-type tumor.

Age is related with unfavorable neuropathological prognostic factors in case of LGG. The higher incidence of anaplasia in older patients results in worse PFS^26,29^. Patients with IDH wild-type and retained nuclear ATRX expression are significantly older, which is concordant and well-known according to the literature^30–33^ and this neuropathological profile is associated with aggressive behavior^34–38^. Similar findings were reported for glioblastoma, where older patients showed less frequently IDH mutation and consequently worse clinical outcome^39,40^. Moreover, this data is concordant to the fact, that patients with IDH wild-type LGG have an increased risk of malignant transformation^41,42^.

Positive CE in MRI is usually interpreted as prognostically unfavorable feature^43,44^. We showed, that age-dependent higher CE rates occur only in case of IDH wild-type glioma: that is a further evidence of the added aggressiveness of these tumors in older population. Thus, it is possible, that the relationship between age and CE is driven by the underlying relationship between age and IDH status.

If a patient was eligible for surgery, it was possible to provide the same extent of resection independently from age. Perioperative complication rates did not differ within elderly people as reported before^45^. Thus, neuro-oncological surgeons should aim for a radical resection for these patients as well^9^, as it provides more beneficial outcome according the actual guidelines^9,10^ and comparative studies^46^. However, the hypothesis that older patients with LGG are undertreated has already been suggested in other studies^47^. According to routine clinical standards, patients are not undertreated in our institution only due to their higher age.

The prognostic unfavorable molecular features consequently led to decreased PFS. We were able to show a predictable association between age and PFS, which could be a great help for physicians in estimating of the expectancies and in treatment suggesting: according to our data one year of life shortens the PFS by 9 days. Thus, a patient with 40 years of age had one year more calculated PFS compared to a patient twice that age. Furthermore, this association was present only in case of IDH wild-type gliomas according to sub-group analysis. Thus, even if there is dependency between older age and less favorable molecular characteristics, older age still imparts a poor prognosis in the IDH wild-type population. The worse PFS corresponded to a significantly worse OS of the older patients as well.

Our study has limitations. The survival endpoints were not reached for all patients, which could limit Cox regression analysis of OS in IDH sub-groups. We did not consider the pre-existing diseases, which were not recorded in our prospective database and could not be gained retrospectively keeping acceptable quality of data. However, patients were processed in our study only if they underwent substantial resection. That includes only patients who were eligible for intracranial surgery with or without general anesthesia. Thus, critically ill patients were indirectly excluded and did not influence our results. The sub-group analysis was restricted in our study due to limited number of cases. Survival analysis requires external validation.

## Conclusions

Age truly matters in LGG. Distinct tumor features like wild-type IDH, malignant behavior and dismal prognosis were more unfavorable in older population, even if the extent of resection was similar. We were able to compute a PFS reduction for 9 days with each year of patient’s life. However, greater age imparted a poorer PFS and higher CE rate only in the IDH wild-type population. That needs to be evaluated in a large prospective cohort.

## Data Availability

The raw data was generated in authors’ institution. The data that support the findings of this study are available on reasonable request from the corresponding author. The data are not publicly available due their containing information that could compromise the privacy of research participants.

## References

[1] Ostrom QT (2020). CBTRUS statistical report: Primary brain and other central nervous system tumors diagnosed in the United States in 2013–2017. Neuro Oncol..

[2] Rasmussen BK (2017). Epidemiology of glioma: Clinical characteristics, symptoms, and predictors of glioma patients grade I-IV in the the Danish Neuro-Oncology Registry. J. Neurooncol..

[3] Ostrom QT (2019). CBTRUS statistical report: Primary brain and other central nervous system tumors diagnosed in the United States in 2012–2016. Neuro Oncol..

[4] Furnari FB (2007). Malignant astrocytic glioma: Genetics, biology, and paths to treatment. Genes Dev..

[5] Louis DN (2016). The 2016 World Health Organization classification of tumors of the central nervous system: A summary. Acta Neuropathol..

[6] Hartmann C (2010). Patients with IDH1 wild type anaplastic astrocytomas exhibit worse prognosis than IDH1-mutated glioblastomas, and IDH1 mutation status accounts for the unfavorable prognostic effect of higher age: Implications for classification of gliomas. Acta Neuropathol..

[7] Magnetic resonance imaging (MRI) exams. OECD. 10.1787/1d89353f-en (2018)

[8] EUROPOP2019 population projections. Eurostat. Vol. TPS00200. https://ec.europa.eu/eurostat/databrowser/view/tps00200/default/table?lang=de (2019)

[9] Weller M (2020). EANO guidelines on the diagnosis and treatment of diffuse gliomas of adulthood. Nat. Rev. Clin. Oncol..

[10] Stupp R (2014). High-grade glioma: ESMO Clinical Practice Guidelines for diagnosis, treatment and follow-up. Ann. Oncol..

[11] Stupp R (2005). Radiotherapy plus concomitant and adjuvant temozolomide for glioblastoma. N. Engl. J. Med..

[12] Morshed RA (2019). Molecular features and clinical outcomes in surgically treated low-grade diffuse gliomas in patients over the age of 60. J. Neurooncol..

[13] Corell A, Carstam L, Smits A, Henriksson R, Jakola AS (2018). Age and surgical outcome of low-grade glioma in Sweden. Acta Neurol. Scand..

[14] Schomas DA, Laack NN, Brown PD (2009). Low-grade gliomas in older patients: Long-term follow-up from Mayo Clinic. Cancer.

[15] Iwamoto FM (2009). Prognosis and patterns of care in elderly patients with glioma. Cancer.

[16] Pouratian N (2008). Low-grade gliomas in older patients: A retrospective analysis of prognostic factors. J. Neurooncol..

[17] Chiu CT (2019). Living arrangements and disability-free life expectancy in the United States. PLoS ONE.

[18] Crimmins EM, Zhang Y, Saito Y (2016). Trends over 4 decades in disability-free life expectancy in the United States. Am. J. Public Health.

[19] Freyschlag CF (2018). Imaging practice in low-grade gliomas among European specialized centers and proposal for a minimum core of imaging. J. Neurooncol..

[20] Yushkevich PA (2019). User-guided segmentation of multi-modality medical imaging datasets with ITK-SNAP. Neuroinformatics.

[21] Yushkevich PA (2006). User-guided 3D active contour segmentation of anatomical structures: Significantly improved efficiency and reliability. Neuroimage.

[22] Daumas-Duport C, Scheithauer B, O'Fallon J, Kelly P (1988). Grading of astrocytomas. A simple and reproducible method. Cancer.

[23] Louis, D. N., Ohgaki, H., Wiestler, O. & Cavenee, W. K. *WHO Classification of Tumours of the Central Nervous System*. Revised 4th Edition edn, Vol. 1 (International Agency for Research on Cancer, 2016).

[24] Stupp R (2017). Effect of tumor-treating fields plus maintenance temozolomide vs maintenance temozolomide alone on survival in patients with glioblastoma: A randomized clinical trial. JAMA.

[25] Weller M (2014). EANO guideline for the diagnosis and treatment of anaplastic gliomas and glioblastoma. Lancet Oncol..

[26] Wick W (2016). Long-term analysis of the NOA-04 randomized phase III trial of sequential radiochemotherapy of anaplastic glioma with PCV or temozolomide. Neuro Oncol..

[27] Holm S (1979). A simple sequentially rejective multiple test procedure. Scand. J. Stat..

[28] Bender R, Lange S (1999). Multiple test procedures other than Bonferroni’s deserve wider use. BMJ.

[29] Nahed BV (2015). Management of patients with recurrence of diffuse low grade glioma: A systematic review and evidence-based clinical practice guideline. J. Neurooncol..

[30] Cancer Genome Atlas Research, N. *et al.* Comprehensive, Integrative Genomic Analysis of Diffuse Lower-Grade Gliomas. *N. Engl. J. Med.* **372**, 2481–2498. 10.1056/NEJMoa1402121 (2015). 10.1056/NEJMoa1402121 PMC4530011 26061751

[31] Ceccarelli M (2016). Molecular profiling reveals biologically discrete subsets and pathways of progression in diffuse glioma. Cell.

[32] Eckel-Passow JE (2015). Glioma groups based on 1p/19q, IDH, and TERT promoter mutations in tumors. N. Engl. J. Med..

[33] Yan H (2009). IDH1 and IDH2 mutations in gliomas. N. Engl. J. Med..

[34] Cohen AL, Holmen SL, Colman H (2013). IDH1 and IDH2 mutations in gliomas. Curr. Neurol. Neurosci. Rep..

[35] Sanson M (2009). Isocitrate dehydrogenase 1 codon 132 mutation is an important prognostic biomarker in gliomas. J. Clin. Oncol..

[36] Leeper HE (2015). IDH mutation, 1p19q codeletion and ATRX loss in WHO grade II gliomas. Oncotarget.

[37] Nandakumar P, Mansouri A, Das S (2017). The Role of ATRX in Glioma Biology. Front. Oncol..

[38] Wiestler B (2013). ATRX loss refines the classification of anaplastic gliomas and identifies a subgroup of IDH mutant astrocytic tumors with better prognosis. Acta Neuropathol..

[39] Aldape K, Zadeh G, Mansouri S, Reifenberger G, von Deimling A (2015). Glioblastoma: Pathology, molecular mechanisms and markers. Acta Neuropathol..

[40] Wirsching HG, Galanis E, Weller M (2016). Glioblastoma. Handb. Clin. Neurol..

[41] Tom MC (2019). Malignant transformation of molecularly classified adult low-grade glioma. Int. J. Radiat. Oncol. Biol. Phys..

[42] Jansen E (2019). Observation after surgery for low grade glioma: Long-term outcome in the light of the 2016 WHO classification. J. Neurooncol..

[43] Montagne A, Toga AW, Zlokovic BV (2016). Blood-brain barrier permeability and gadolinium: benefits and potential pitfalls in research. JAMA Neurol..

[44] Pallud J (2009). Prognostic significance of imaging contrast enhancement for WHO grade II gliomas. Neuro Oncol..

[45] Capelle L (2013). Spontaneous and therapeutic prognostic factors in adult hemispheric World Health Organization Grade II gliomas: A series of 1097 cases: Clinical article. J. Neurosurg..

[46] Jakola AS (2012). Comparison of a strategy favoring early surgical resection vs a strategy favoring watchful waiting in low-grade gliomas. JAMA.

[47] Kaloshi G (2009). Supratentorial low-grade gliomas in older patients. Neurology.

